# Nanotechnology-Aided Advancement in Combating the Cancer Metastasis

**DOI:** 10.3390/ph16060899

**Published:** 2023-06-19

**Authors:** Arun Kumar Singh, Rishabha Malviya, Bhupendra Prajapati, Sudarshan Singh, Deepika Yadav, Arvind Kumar

**Affiliations:** 1 Department of Pharmacy, School of Medical and Allied Sciences, Galgotias University, Greater Noida 203201, India; arunthakur01996@gmail.com (A.K.S.); deepika.21smas2020010@galgotiasuniversity.edu.in (D.Y.); 2 Shree S. K. Patel College of Pharmaceutical Education and Research, Ganpat University, Kherva 384012, India; 3 Department of Pharmaceutical Sciences, Faculty of Pharmacy, Chiang Mai University, Chiang Mai 50200, Thailand; 4 Chandigarh Engineering College, Jhanjeri, Mohali 140307, India; arvindkumar.j1753@cgc.ac.in

**Keywords:** cancer, targeted therapy, nanotechnology, patient care, chemotherapy, cancer metastasis

## Abstract

Modern medicine has been working to find a cure for cancer for almost a century, but thus far, they have not been very successful. Although cancer treatment has come a long way, more work has to be carried out to boost specificity and reduce systemic toxicity. The diagnostic industry is on the cusp of a technological revolution, and early diagnosis is essential for improving prognostic outlook and patient quality of life. In recent years, nanotechnology’s use has expanded, demonstrating its efficacy in enhancing fields such as cancer treatment, radiation therapy, diagnostics, and imaging. Applications for nanomaterials are diverse, ranging from enhanced radiation adjuvants to more sensitive early detection instruments. Cancer, particularly when it has spread beyond the original site of cancer, is notoriously tough to combat. Many people die from metastatic cancer, which is why it remains a huge issue. Cancer cells go through a sequence of events known as the “metastatic cascade” throughout metastasis, which may be used to build anti-metastatic therapeutic techniques. Conventional treatments and diagnostics for metastasis have their drawbacks and hurdles that must be overcome. In this contribution, we explore in-depth the potential benefits that nanotechnology-aided methods might offer to the detection and treatment of metastatic illness, either alone or in conjunction with currently available conventional procedures. Anti-metastatic drugs, which can prevent or slow the spread of cancer throughout the body, can be more precisely targeted and developed with the help of nanotechnology. Furthermore, we talk about how nanotechnology is being applied to the treatment of patients with cancer metastases.

## 1. Introduction

Metastasis occurs when cancer cells from a primary tumour travel to neighbouring tissue or a distant site, where they can grow into secondary or tertiary tumours. Even though cancer metastasis causes a great deal of suffering and death, its exact mechanisms have not yet been fully elucidated. The ability of metastatic cells to spread to different organs is just one of the many reasons why current metastatic cancer therapies have been so unsuccessful; another is the tumour cells’ heterogeneity, plasticity, and unique tumour microenvironment (TME) complexity, each of which may respond differently to the treatment [1,2]. The metastatic process includes a chain reaction of cellular biological processes known as metastatic cascades. Malignant cells lose their capacity to adhere to one another at the location of the initial tumour, allowing them to divide and penetrate the vasculature. Intravasation into blood arteries and subsequent adhesion to artery walls may facilitate this cell type’s dissemination across the circulatory system (attachment). Tumour nodules and deposits, both micro- and macrometastatic [3], originate from invasion, the creation of pre-metastatic niches, colonisation, and multiplication of cancer cells in a new environment. Epithelial stem cells and differentiated epithelial cells undergo epithelial-to-mesenchymal transition (EMT) throughout the metastatic cascade, changing into tumour cells with mesenchymal features that are good at avoiding the immune response [4]. Transcoelomic dissemination occurs when cancer cells that started in the digestive system spread to other areas of the body.

Evidence that the distant secondary milieu may be primed and made ready for future metastatic development before the arrival of cancer cells gave rise to the idea of the pre-metastatic niche. Pre-metastatic niche formation has been characterised as a time-dependent series of processes that precede the arrival of cancer cells to the site of illness, effectively preparing the location for the engraftment and colonisation of metastasising tumour cells. Because cancer cells leave the main tumour with their destination well specified, the development of the pre-metastatic niche strongly suggests that metastasis to a specific organ is not a random event. It is unclear whether the circulating tumour cells actively seek out these locations or just take advantage of the favourable conditions present in these pre-metastatic habitats [5]. Non-malignant bone marrow-derived haematopoietic progenitor cells (BMDCs) expressing the vascular endothelial growth factor receptor-1 (VEGFR-1) precede the arrival of even single metastatic tumour cells and macro-metastatic disease at distant sites, according to early work by Kaplan and colleagues defining the pre-metastatic niche and its temporal and functional relationship to metastasis. Finally, they showed that cancer-specific factors elevated VLA-4 ligand and fibronectin at distant pre-metastatic locations, which recruited PDGFR-expressing cells and resulted in the establishment of favourable niches for arriving tumour cells. These findings were the first to suggest that non-neoplastic host cells might be able to pre-define a metastasis site. However, subsequent research indicated that while recruitment of VEGFR-1+ BMDCs enhances metastasis in some cases, independent non-VEGFR-1-mediated mechanisms of spontaneous metastasis might also play a role. Hiraksuka et al. demonstrated that MMP9 expression in endothelial cells and macrophages in the pulmonary pre-metastatic niche, in conjunction with the secretion of VEGF-A, TNF-, TGF-, and the inflammatory chemo-attractants S100A8 and S100A9, drives recruitment of CD11b+ (Mac1+) myeloid cells to the pre-metastatic milieu. More recent research has demonstrated that S100A8 and S100A9 promote chemoattractant production and pulmonary metastasis by stimulating NF-kB via Toll-like receptors [6]. This, in turn, drives the expression of serum amyloid 3A in the pre-metastatic lung. Concurrently, we found that lysyl oxidase (LOX) plays a significant role in the development of pre-metastatic niches in the lungs, liver, and brain. LOX was shown to be co-localised with fibronectin in sites of future metastasis, where it facilitated matrix remodelling, enhanced CD11b+ BMDC recruitment, and increased metastasis after being secreted from the hypoxic tumour environment of primary breast tumours. It has been established that blocking the recruitment of BMDCs to pre-metastatic locations is sufficient to halt the creation of niches and the spread of metastases by either antibody therapy or depletion. Previous research has shown that LOX interacts with fibronectin to regulate its activity, suggesting that the initial deposition of fibronectin and LOX during pre-metastatic niche formation significantly contributes to the generation of suitable microenvironments that facilitate the recruitment of BMDCs and, ultimately, the successful colonisation by tumour cells. Factors produced by melanoma cells seem to stimulate hepatic stellate cells to a myofibroblast-like phenotype linked with alpha-smooth muscle actin expression and cytoskeletal alterations, hence establishing a growth-supportive environment and colonisation of arriving cancer cells, as seen in investigations of hepatic metastasis.

Prostate cancer bone metastases rely on haematopoietic cells much as they do in the lungs to facilitate tumour cell colonisation of secondary organs. Prostate cancer cells thrive in the complex, multicellular milieu of the bone, making it an ideal site for the development of skeletal metastases. The increased production of CCL2, IL-6, and VEGF by CD11b+ cells in the bone marrow has been linked to the metastatic spread of prostate cancer. It is not unexpected that many malignancies exhibit a deadly predisposition to form inside the bone and bone marrow since there is a large overlap in the molecular machinery between metastasising tumour cells and haematopoietic cells. This same homing behaviour has been demonstrated to be mediated by the CXCL12/SDF1-CXCR4 axis, which is also used by osteoblasts and bone marrow stromal cells to recruit HSCs. Several osteotrophic malignancies, including breast, ovarian, prostate, and neuroblastoma, are believed to metastasise to bone in an SDF-1-dependent manner due to their high expression of the CXCR4 receptor [7]. Recently, it has been shown that prostate cancer cells may directly compete with HSCs for occupancy of the bone marrow niche, ‘hi-jacking’ and drastically modifying the microenvironment to encourage the production of bone metastases and drive HSCs into the peripheral blood pools. However, whether the appropriation of the bone marrow niche happens before cancer cell entrance, as found in pulmonary pre-metastatic niche creation, is still unknown, partly owing to experimental restrictions preventing such research.

Metastasis is influenced by both the initial tumour at the site of metastasis (“seed”) and the competence of the distal organ (“soil”) [8,9]. Thus, the “initial site,” the vasculature surrounding the tumour, and the tendency of the disease to spread to certain organs are the determining factors in the most common sites of metastasis. Cell polarity shifts, cytoskeleton rearrangement, mutation uptake, post-translational changes, and the synthesis of membrane proteins are all part of this metastatic cascade [10].

Metastatic cancer cells are notoriously nocturnal and will not be discovered until the tumour has metastasised and spread too far. As a result, it becomes more challenging to both diagnose and treat metastatic cancer after it has spread. The metastatic process can have begun in some patients years before the initial disease was detected. Detecting metastatic breast cancer in its early stages may be difficult since symptoms may not present for years. Metastases from lung cancer, on the other hand, may already be present in several organs before the patient even realises anything is wrong. If the primary tumour is too small to see, the challenge of treating metastatic illness grows considerably (known as “cancer of unknown origin”). Some metastases may be hard to attribute to the primary tumour because they seem so different. Even though metastases of kidney and breast cancer do share many qualities with their respective primary tumours, many metastases do not have the same characteristics as the original tumour. You may learn more about the pathophysiology, methods, and issues associated with metastasis by reading the aforementioned articles [11,12,13]. Nanomedicine, the use of nanotechnology in healthcare, is a relatively new area that has been receiving a lot of attention recently. Its success may be attributed to the interdisciplinary nature of the subject itself, which combines knowledge from fields as disparate as physics and chemistry for the synthesis and characterisation of nanomaterials (NMs) with knowledge from biology and medicine for their functional applications. The unusual features of NMs make them well suited to study in a broad range of biological applications, even though they have been primarily studied in the electrical and industrial domains. This has led to a slew of research into how NMs interact with their surroundings in the body, with the ultimate goal of pinpointing which properties of NMs cause specific responses. Research groups have been able to exploit the various unique properties of NMs to improve therapeutic and diagnostic outcomes in cancer research and clinical use as a result of our increased understanding of bionano interactions in combination with the rapid developments and in-depth knowledge gained in several medical fields such as oncology. NMs’ special properties result from their many distinguishing features, such as their large surface area relative to their volume, their scalability, and the availability of a wide variety of materials, some of which exhibit additional peculiarities, such as the superparamagnetism of magnetic NMs and the surface plasmon resonance (SPR) of gold NMs.

The biodistribution and clearance of NMs are also greatly affected by the ease with which their surface may be functionalised in a variety of ways. Finally, a nanocarrier with numerous features and functions may be engineered by incorporating diverse capabilities into a single entity (i.e., a fluorescence probe, drug, or antibody attached to a magnetic carrier). Several NMs have been licenced for clinical use in cancer treatment and diagnostics, and many more are in the midst of clinical studies. There are two main types of NMs used in medicine and diagnosis: organic NMs (such as liposomes, polymeric NMs, micelles, etc.) and inorganic NMs (such as iron oxide, gold, silica, etc.). Organic NMs are being developed for applications including vaccination, long-lasting depot delivery systems, hemostasis, and topical agents for systemic delivery through the skin because of their high biocompatibility and reduced long-term side effects. Both the administration of small molecule medications for the treatment of cancer (such as breast, melanoma, head and neck, etc.) and gene therapy are primary targets for organic NMs administered intravenously. Although inorganic NMs have shown great promise in preclinical studies, their translation into clinical practice has been slower due to concerns about their safety and long-term deposition in organs such as the liver and spleen and a lack of knowledge and consensus on these issues. Clinical experiments are now being conducted on the use of inorganic NMs for a variety of purposes, including thermal ablation of tumours and intraoperative sentinel lymph node imaging, although their primary usage remains as MRI contrast agents [14].

Despite many people working hard to find better cancer treatments, it is still the second biggest cause of death globally. Recurrence rates are significant because distant tumours are so difficult to remove. The prognosis for individuals with non-localised tumours is often dismal, even though surgical intervention, radiation treatment, and chemotherapy are generally successful at extending the lives of patients with localised tumours [15]. Therefore, more than 90% of cancer-related fatalities are caused by tumours that have migrated to distant organs. Limiting metastasis in individuals treated for localised illness and limiting recurrence are two of the largest difficulties in clinical cancer therapy. To overcome these obstacles, we need to find more efficient ways to eradicate tumour cells systemically. Unfortunately, little is known about the cellular and molecular pathways that control metastasis. It is now understood that both the main tumour site and metastatic locations entail a complicated interaction between cancer cells, immune cells, and stromal cells. Tumour cells may use a wide variety of techniques to evade regular immune monitoring, making immune evasion the essential phase in tumour growth from an immunological point of view. In particular, tumour growth and metastasis are promoted by intratumoral inflammation, the infiltration of multiple host immune cell types (most notably tumour-associated macrophages [TAMs]), and the secretion of various cytokines and growth factors by cancer cells or immune cells. Tumours’ adoption of immunological features works in tandem with these features to facilitate tumour cell metastasis and colonisation beyond the initial tumour location.

Immunotherapy has emerged as a vital tool in the battle against some forms of cancer in recent decades. Immunotherapy has evolved into an effective regimen for cancer patients either on its own or in conjunction with other treatments such as surgery, chemotherapy, and radiation therapy by bolstering the host immune responses against tumours, supplying modified immune system components, or counteracting signals produced by cancer cells that suppress immune responses. As our knowledge of the immune system expands rapidly, more and more approaches are being taken to modulate the immune response as a means of treating cancer, including the use of small compounds, peptides, recombinant antibodies, vaccinations, and cellular therapy modalities. The use of immune checkpoint inhibitors and cell treatments, in particular, have shown to be very effective against cancer thanks to these immunotherapies. We solicited submissions of original publications, case reports, clinical investigations, and reviews on the topic of anti-tumour immunotherapy to showcase the growth and variety of this discipline. The benefits and drawbacks of using microbes to treat cancer are discussed by Ukasiewicz and Fol in this Special Issue. It is possible that microorganisms, or a component of them, might trigger the immune system to remove cancer cells. Microorganisms have the potential to be engineered into high-functioning delivery vehicles. Microorganisms have great potential, but their widespread use is now hampered by factors such as the presence of an accompanying infection and the restricted variety of cancer options [16].

In patients with lung cancer, Arai et al. found that as lipid buildup increased, the quantity and activity of peripheral blood dendritic cells (DCs) declined. Initiating a targeted anti-tumour immune response requires DCs to transmit antigen peptides to T cells. The loss in APC number and function in cancer development and metastasis was induced by the buildup of aberrant lipids in DC. This might provide researchers with a fresh avenue to explore while looking for novel cancer treatments.

For their research on anti-tumour immunity, Rollins and Gibbons Johnson zeroed their attention on PD-L1. Recent breakthroughs in many forms of cancer have promoted a new discipline called checkpoint-associated anticancer treatment, which may one day be able to completely eradicate cancer. Activated CD8+ T cells fared better in the absence of CD80 expression, one of the PD-L1 ligands, as shown by the research team led by Johnson and published in this Special Issue. It highlights CD80’s significance in checkpoint blockade for anticancer therapy development and execution [17].

The field of cell treatment, which has tremendous potential, has also advanced rapidly in recent years. Despite the advent of more sophisticated treatments such as CAR-T and TCR-T, the original tumour-infiltrating lymphocyte (TIL) therapy has shown promising results due to its lengthy history and low risk of side effects. Li et al. conducted a clinical trial showing that TIL combination with IFN-alpha treatment significantly improved disease-free survival and overall survival rates in patients with malignant melanoma compared to no cell therapy [18].

Due to its essential involvement in regulating genes involved in cell proliferation and metabolism, cereblon is a pivotal protein in autosomal recessive nonsyndromic mental retardation and metabolic disorders. It plays a variety of functions in the treatment of cancer patients with immunomodulatory drugs. The many roles and processes in the implementation of immunomodulatory medications are summarised by Shi and Chen in this Special Issue, which might be of tremendous help to any immunomodulatory drug. Although there is much more to be said on the subject of cancer immunotherapy, the papers in this Special Issue cover a wide range of research methods, clinical investigations, and systematic reviews. Our goal in compiling this Special Issue was to facilitate improved implementation of immunotherapy for cancer patients by providing useful information to researchers and physicians [19].

The goal of immunotherapy for solid tumours is to eradicate the primary tumour, destroy distant metastases, and prevent recurrence by stimulating the innate immune system to mount a systemic anti-tumour immune response. There has been an impressive clinical success with the immunotherapy model. Particularly popular treatments are those that use chimeric antigen receptor (CAR) T cells or immune checkpoint blockade, which involves the elimination of immune-inhibitory receptors such as programmed cell death protein 1 (PD-1) or cytotoxic T lymphocyte antigen-4 (CTLA-4) [16]. Unfortunately, insufficient immune responses have rendered current clinical immunotherapies ineffective for some patients and some types of cancer. This is especially true when immunologically “cold” solid tumours generate an immunosuppressive microenvironment that prevents the host immune system from attacking cancer cells [20].

Currently, there is a wide variety of cancer therapy medications available for the treatment of metastasis; nevertheless, the greatest difficulties lay in the detection and evaluation of metastasis, as well as the most effective targeting and drug administration strategies. As the same medications are often used to treat both primary and metastatic tumours, the therapy of metastases is difficult and inefficient. Surgery, chemotherapy, immunotherapy, radiation therapy, and palliative care are all common approaches to treating metastatic cancer; however, these treatments have their limitations and may only benefit a small number of patients. Due to their nonspecific distribution and cytotoxicity towards cancer cells and normal cells, conventional chemotherapy and radiation are less effective and have more unfavourable side effects [21].

Concerns have been raised about the proliferation of stem-like cells and the present state of chemotherapy. These issues include a lack of therapeutic specificity, heterogeneity, a long half-life, excellent solubility, and resistance to several medicines. Innovative diagnostic and therapeutic approaches are required to prevent or reverse metastatic illness (metastatic disease), secondary tumour formation, and/or underlying metastatic processes to diminish the restrictions and bottlenecks of present medicines. By controlling materials at the nanoscale, it is possible to build devices, systems, and tools with potential medicinal and other uses. The unique physical and chemical features of nanoparticles (NPs) enable individualised, programmable, and flexible therapeutic and theranostics uses. Nanomedicine, along with other nanomaterial-based approaches, may have a profound impact on cancer detection and therapy. Cancer therapeutics based on nanotechnology [22,23] may one day make standard treatment techniques such as chemotherapy less dangerous. Historically, nanomedicines have been used to better target the delivery of systemically given chemotherapeutic treatments by altering biodistribution and accumulation at the target region. Using bio-functionalised NPs loaded with medications that circumvent biological barriers to promote effectiveness [24] may improve the transport and cellular translocation efficiency of therapeutic molecules while reducing morbidity. Attaching a delivery carrier with an affinity for different surface receptor proteins to the cellular wall is one way in which a highly sensitive multi-modal nano-bio molecule might transport receptor-specific nanocarriers to the target tissue [25]. Several nanomedicine products have received regulatory approval and are now in clinical usage in the United States and elsewhere, while many more are currently being studied in preclinical and clinical settings to treat various types of cancer and metastatic indications. While the supplementary material highlights ongoing clinical research into nanotechnology’s potential as a cancer therapeutic and diagnostic tool, Table 1 gives a review of approved nanotechnology-based cancer medicines. The next section of this review delves deeply into the potential medical uses of nanotechnology. Nanomedicine therapeutic development for cancer (metastasis) has been the subject of various publications. Additional information on the cancer nano-drugs described above may be found in works [26].

**Figure 1:**
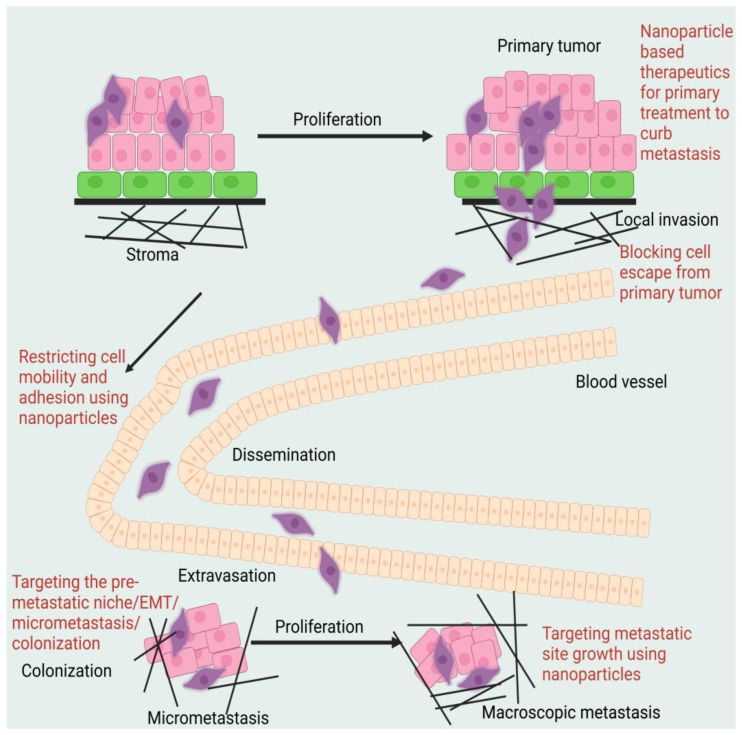
Pictorial representation of metastatic cascade.

A large portion of medical research in recent decades has aimed at creating drugs that specifically target cancer cells rather than normal cells. Targeted treatment has allowed for significant progress in precision medicine, but it comes with several undesirable side effects, and drug resistance remains a persistent issue [27]. Since its rise to the number two spot, cancer has been met with insufficient treatment options. As a result, more resources are being devoted to finding ways to treat cancer and combat the side effects of existing drugs. In this review, the authors will outline the benefits of using nanotechnology in conjunction with traditional (or other) approaches and techniques for treating metastatic malignancies, with a special emphasis on nanotechnology-based medications and diagnostics (see Figure 1). Preclinical research is the primary topic of this debate. Although nanotechnology shows promise in the fight against cancer metastasis, most ongoing studies are still in the pre-clinical phase. Keeping in mind that discovering therapeutically effective therapies for metastasis is a difficult task provides some background for why this problem is so difficult to tackle [28]. Looking at the search-NIH grant database, for example, the authors see that just 10% of cancer metastasis awards in the previous five years were allocated to clinical efforts, whereas 20% of total cancer money was allocated to such projects.

All treatment methods and technologies, including nanotechnology, are progressing at a glacial rate when it comes to the creation of effective anti-metastatic drugs [29,30]. In the context of cancer treatment, the authors investigate the potential of a unique combination of these nanotechnology breakthroughs with a variety of tailored delivery techniques to attack cancer metastasis at different phases of the metastatic cascade. These data will help us figure out how optimum designs of nano-methods interact with the biology of metastasis and what kind of results the researcher may expect. Strategies to address the potential hazards of deploying nanotechnology and more conventional approaches are also in the works [31]. In the end, the author will discuss the most recent developments, challenges, and perspectives in the utilisation of collaborative efforts to halt the spread of cancer. This review examines the status of nanotechnology translation into patient care and the challenges that must be overcome to expedite the usage of these technologies. To impede intracellular signal transmission and target epidermal growth factor (EGFR) receptors, the new monoclonal antibody cetuximab was introduced to the market in 2003. mCRC, which is resistant to chemotherapy with irinotecan-based medications, is the principal indication listed in its medical insert (Merck, Germany) [32]. According to earlier research, mCRC often coexists with genetic alterations such as K-Ras mutations, and the mutant mCRC is no longer controlled by EGFR. The EGFR-targeting medications will thereafter lose their efficacy. As a result, K-Ras wild-type mCRC is the only condition for which EGFR inhibitors are appropriate. The current standard regimen for the first-line treatment of patients with RAS wild-type mCRC uses cetuximab in conjunction with chemotherapy. In patients with wild-type RAS mCRC, particularly those with initial lesions on the left, cetuximab substantially increases objective response rate (ORR), progression-free survival (PFS), and overall survival (OS). However, even in individuals with left-sided colorectal cancer, where cetuximab’s effectiveness predominates, roughly 30% of patients experience failure. Additionally, the duration of its therapeutic use is not very long. Cetuximab clinical trial outcomes for the treatment of mCRC in recent years have also been erratic [33]. On its effectiveness and negative effects, there is now no unambiguous consensus.Table 2 shows the advantages and disadvantages of different nanostructures. This paper employed a meta-analysis together with recently released data to evaluate the effectiveness of cetuximab paired with chemotherapy for the treatment of mCRC in order to produce a more trustworthy evidence-based medical proof for its clinical usage [34].

## 2. Metastasis Suppression through Primary Tumour Targeting with Nanoparticles

**Figure 2:**
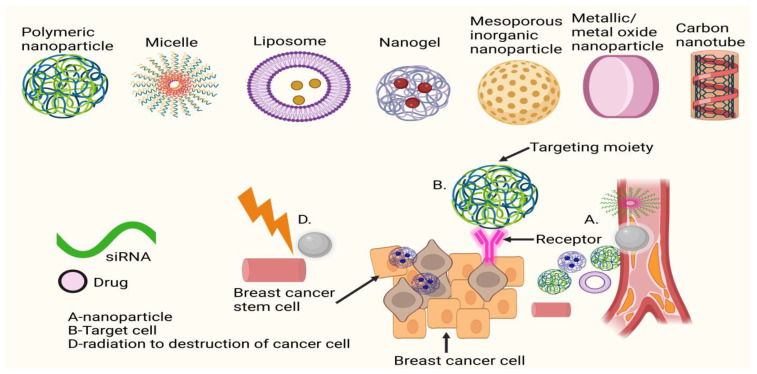
Schematic representation of the mechanism of action of nanoparticle-assisted targeting of primary cancer to mitigate metastasis.

Anti-tumour measures, such as inducing tumour cell apoptosis, targeting cancer stem cells (CSCs), preventing EMT, regulating the tumour microenvironment (TME), and stimulating the immune system, have been shown in numerous studies to be effective in preventing tumour spread using NPs to target the primary cancer site. In preclinical studies, nano liposomal ceramide formulations have shown promise as a treatment for tumour metastasis. C6 ceramide liposomes delay or eliminate highly aggressive breast cancer cells that have metastasised [35]. Podophyllotoxin (PPT) may help with lung cancer at any stage. Nano-delivery systems made of layered double hydroxides (LDH) have been used to collect PPT because of their increased effectiveness and lower toxicity in mice. Harrison et al. identified a miRNA/circRNA regulatory axis that promoted the metastasis of lung squamous cell carcinoma via CDR1-mediated modulation of Golgi trafficking. Lipid nanoparticle (NP) delivery of miR-671-5p to the noncoding CDR1as/CDR1 axis suppressed lymphatic metastases in mice in vivo. Xu et al. [36] developed a systemic nanotherapeutic (H@CaPP) that combines the anti-inflammatory medication piceatannol with the anti-thrombotic agent low molecular weight heparin to halt tumour development at any stage. The adoption of this nanoformulation made it more difficult to suppress EMT, limit the spread of microthrombi, and prevent the formation of a pre-metastatic niche. When this nanoformulation was administered to mice with breast tumours alongside chemotherapy or surgical resection, the mice’s survival time greatly improved. To treat patients with metastatic colon cancer and squamous cell carcinoma of the head and neck, the FDA authorised the use of the anti-EGFR monoclonal antibody cetuximab. It was examined in conjunction with the study of convection-enhanced transport of multifunctional magnetic iron-oxide nanoparticles (IONPs) to glioblastoma multiforme (GBM). Cetuximab-IONP bound/internalised GBM cells more efficiently than other EGFR-targeted drugs, resulting in increased longevity and the capacity to halt tumour recurrence or metastasis. Figure 2 shows the diagrammatic representation of the mechanism of action of nanoparticle-assisted targeting of primary cancer to mitigate metastasis.

Studies have shown that effective distribution and target-reaching depend on the treatment’s circulation time being prolonged using therapeutic nanoparticles of the appropriate size. Evidence from multiple studies [37] suggests that combining nanotechnology with chemotherapy and other medicines could improve treatment outcomes for cancer. In order to achieve CD44-targeted drug delivery to breast cancer, the group led by Liu et al. developed a CBSA-protected gold nanocluster (AuNC) of the proper size and shape. This type of treatment accelerated the spread of breast cancer that had begun subcutaneously. Next, the researcher threw in the nitric oxide (NO) donor to promote tumour development by increasing oxygen and nutrients being delivered to the tumour. In contrast, nanoparticles loaded with paclitaxel and indocyanine green (ICG) have been found to significantly reduce primary tumour growth and prevent lung metastasis.

Concerns about tumour size, medication administration, and toxicity are all addressed by this theranostic strategy. Combining the tumour-homing peptide iRGD with the multistage responsive NPs dendrite graft l-lysine (DOX) and indocyanine (IDD), with NO donor-modified HA shell (HN) and laser radiation may result in greater NP accumulation in the tumour [38]. After standard chemotherapy has failed, ceramide nanoliposomes may be effective in treating metastatic melanoma and breast cancer. When used with Sorafenib, it has a synergistic impact on tumour development and metastasis in mice. 5-fluorouracil (5-FU), one of the most widely used chemotherapeutic medications, may be delivered via LDH and has been demonstrated to inhibit the proliferation of colon cancer cells. The use of NPs that target the primary TME to prevent and cure cancer metastases has great potential. Because low oxygen levels are associated with tumour resistance and metastasis [39], several therapies, such as radiation, phototherapy, and vascular-disrupting medicines, attempt to increase oxygen levels in the cancer microenvironment. It has been established that the mTOR inhibitor temsirolimus decreases angiogenesis by blocking HIF-1 from inducing VEGF synthesis [40]. When combined with immunosuppressive medication, such as the chemical combretastatin A4, which attenuates the hypoxic milieu [41], the expression of HIF-1 was substantially decreased, immune suppression was reversed, and lung metastasis rates were reduced.

Several reports suggest that the therapy of cancers, in general, might benefit from a unique nano-combination formulation that eliminates or reduces metastatic tumour masses by focusing on cancer stem cell (CSC) activity. By self-renewing and generating the many subpopulations of cancer cells that comprise a tumour, cancer stem cells (CSCs) have been identified [42]. These CSCs are sometimes referred to as TSCs or TICs. [43] Numerous signalling pathways control the potential for CSCs to undergo differentiation into various malignant phenotypes. These events are driven by the primary tumour to create the conditions for metastasis to occur, including metastasis-propagator cell generation, a pre-metastasis niche, and an organ microenvironment that is friendly to CSC migration. Recent investigations [44,45] have shown the critical function of cancer stem cells (CSCs) in carcinogenesis, EMT, invasion, and metastasis.

CSCs may also contribute to tumour heterogeneity, chemoresistance, and radio resistance [46], and they may be positioned in inaccessible parts of tumours, making it difficult to target them with pharmacological therapy. The central function of CSCs in carcinogenesis, tumour development, and treatment resistance is now well established. Cancer stem cells (CSCs) secrete surface markers that might be targeted by therapeutic antibodies in molecularly targeted treatments for a variety of cancers. Cancer stem cells (CSCs) are a possible target for nanomedicine’s use in cancer and metastasis therapy [47]. As a result of the features of CSCs being identified, many new nano drug formulations have been developed to preferentially target CSCs in tumour tissues. The development of novel nano theranostics (NGNT) for therapeutic and diagnostic use is one such example of CSCs. Multifunctional nanoparticles may be used to locate and treat specific regions by carrying ligands or biomarkers [48]. Drug-carrying nanoparticles administered by stem cell therapy improved anticancer and antimetastatic effects and CSC clearance, in vivo, in mice harbouring breast cancer xenografts via synergistic inhibition of TGF-signaling pathway suppression. The combination of thermotherapy and chemotherapy may be seen in the systemic injection of a chemotherapeutic medicine encapsulated in antibody-coated, silica-based, multifunctional magnetic nanoparticles. Tissue-specific stem cells (CSCs) inhibited lung tumourigenesis and metastasis in mice [49]. There was evidence that CDDP/m nanomedicine might eliminate both undifferentiated and differentiated cancer cell populations in head and neck malignancies by regulating intratumoral and intracellular travel. Patients with advanced or metastatic cancer may benefit from this approach if it is used after cisplatin treatment. Additionally, a cell-differentiation-regulated nanomedicine was investigated in mouse models of breast cancer to see whether it may increase the chemotherapeutic response and break through the therapeutic barrier caused by CSC-derived heterogeneity. Nanoparticles loaded with both all-trans retinoic acid (ATRA) and camptothecin (CPT) have been found to reduce tumour growth, stop tumours from returning after surgery, and stop cancer from spreading to other parts of the body [50,51,52]. Combining the anticancer effects of two medications with distinct mechanisms of action has been demonstrated to be successful, helping patients to overcome intratumour heterogeneity and therapeutic barriers associated with distant metastasis. Liu et al. [53] combined conventional chemotherapy, anti-CSC treatment, and immune checkpoint blockade therapy in a nanodevice with spatiotemporal control to treat metastatic breast cancer in mice. In this preparation, the PD-1/PD-L inhibitor and the drug delivery mechanism (a nanoparticle (NP) with dual sensitivity to enzymes and pH and a micelle/liposome double-layer structure) are combined. It is possible to employ HA-coated liposomes encasing cabazitaxel and silibinin, an inhibitor of cancer stem cells, to target CD44 receptors on cancer stem cells [54].

The cell/process linkage in metastasis was further shown by the findings of Kaushik et al. [55], who discovered that CSCs and EMT are connected and that EMT is responsible for the development of CSCs. Gold nanoparticles coated in polyethene glycol (PEG) and cold plasma were used to treat GBM cells and xenografted animals. The inhibition of tumour growth and metastasis through the PI3K/AKT pathway was seen when both medicines were administered together, even at modest dosages. The reversal of EMT was assisted by the downregulation of CSC hallmarks Slug, ZEB-1, and CD133+ cells. The reversal of EMT after TGF suppression has been linked to an intrinsic anticancer effect of Gadolinium (Gd)-containing metallofullerenol NPs in breast cancer cells (Liu Y. et al. [53]). Reduced numbers of pulmonary and liver micrometastases in living animals proved successful in this endeavour. In hypoxic tumour regions where HIF-1 was suppressed, data revealed a lower CSC population existed. Multiple cancer types, including tumours originating from CSCs, have been found to have EpCAM overexpression. Petersburg et al. [56] developed chemically self-assembled nano-rings (CSANs) to modify the surface of T cells in a way that is not genetic and can be undone (CSANs). The results of this study suggest that PARs T cells may be employed as a supplementary technique of T-cell targeting to eliminate solid tumours that express EpCAM and human CD3 receptors (Epcam/CD3). These bispecific and polyvalent CSANs show promise in vitro and in vivo, suggesting they might be used for cell-directed immunotherapy against cancer and metastasis [57,58]. Treatment of cancer metastasis may be enhanced using nano-immunotherapeutics, which boost the host’s immune defence and anti-tumour immunology response.

Nanoparticles (NPs) may be used to precisely deliver drugs to the site of a tumour, where they can stimulate both local and systemic immune responses [59]. Mica NPs were demonstrated to direct bone marrow-derived macrophages and dendritic cells toward regulating the local microenvironment, thus lowering immunosuppression and increasing the immune system’s ability to fight cancer. Tumour cell growth and subsequent metastasis were slowed because of this change [60]. Mice with breast cancer had an improved host immune response (increased production of pro-inflammatory cytokines and NK cell cytotoxicity) after receiving preventive treatment consisting of oral administration of Selenium (Se) NP-enriched lactobacillus followed by subcutaneous injection [61]. To reduce distant metastasis in mice with melanoma and breast cancer, Rao et al. [62] used a biomimetic synthetic approach and cell membrane-coating nanotechnology. Their research suggests that cell-membrane-coated magnetic nanoparticles (gCM-MNs) altered genetically to disrupt the CD47-SIRP signalling pathway might be used to effectively stimulate macrophages in cancer immunotherapy. Increased cancer cell phagocytosis via macrophages and a stronger anti-tumour T-cell response are both achieved via the MN core’s facilitation of repolarisation of tumour-associated macrophages from M2 to M1. Increased systemic circulation and tumour accumulation of gCM-MNs with less off-target effects are achieved by the biomimetic gCM shell shielding the MN core from immune clearance and the MN core delivering the gCM shell to the TME via magnetic navigation. As a Toll-like receptor 9 (TLR9) agonist, the antigenic peptide CpG-ODN is widely used in cancer immunotherapy to stimulate an effective immune response against tumours. Boosting the anticancer immune response via the use of a combined immunotherapy approach is discussed by Liu and colleagues [63]. Curcumin was enclosed in nanomedicine, a nano vaccine was built out of CpG-ODNs, and cationic polymeric NPs were put into a hydrogel. Tumour-specific T-cell immunity was boosted by the nano vaccine, while cancer cell killing was facilitated by the nanomedicine. In mice, treating postoperative breast cancer and pulmonary metastases with a combination of therapies was effective. When chemotherapy is coupled with immunotherapy and nanotechnology, anti-tumour immunity is dramatically increased.

Nano-assemblies of glutathione-activatable drugs, such as dimer-7-ethyl-10-hydroxy camptothecin (d-SN38) and dimer-lonidamine (d-LND)-coloaded bilirubin NPs (SL@BRNPs), were developed by Yang et al. [64] to boost the immunotherapeutic efficacy of anti-PD-L1 antibody. By increasing the circulation of SL@BRNPs in primary breast cancer and stimulating an efficient anticancer impact and immune response, the iRGD peptide may be able to treat or prevent lung metastasis. In this scenario, medicine administration is improved by linking activatable drug dimers with stimulus-responsive drug release. Kuai et al. [65] stated that nanodiscs that imitate high-density lipoproteins were used to carry DOX into tumours, where it may improve the anti-tumour immune response and allow immune checkpoint blockade. A DOX/indoximod (IND)-liposome construct dramatically decreased liver metastases in colorectal cancer rats when given in combination with PD-L1 therapy. Lu et al. [66] report a novel nano-enabled approach to inhibiting the indoleamine 2,3-dioxygenase (IDO-1) pathway, in which a liposomal carrier is self-assembled from the phospholipid-conjugated prodrug IND. When PD-L1 antibody therapy was added to this DOX/IND-liposome, tumour development was reduced, and pulmonary metastases were eliminated in mice with breast cancer. Li et al. [67] developed nano-DOX to reprogram the immunosuppressive tumour microenvironment (TME) of GBM and to activate an immune response against the malignancy and its metastases. In GBM cells and xenograft models, it was shown that nanodiamonds functionalised with polyglycerol loaded with DOX on their surfaces induced autophagy rather than apoptosis. Checkpoint blockade therapy, photothermal agents, and nanoparticles may all be useful in treating tumours that metastasise. Chen et al. [68] created poly lactic-co-glycolic acid (PLGA) nanoparticles loaded with ICG and imiquimod (R837) and injected them intravenously (IV) into mice to cure breast cancer metastasis (CTLA-4). When used in conjunction with immunoadjuvant nanoparticles and checkpoint inhibition, photothermal therapy has the potential to stimulate the body’s anti-tumour immune response, therefore halting the growth of the primary tumour and any distant tumours or metastases [68].

If your immune system has been stimulated, this will happen. Polydopamine-PEG-R848-CD NP, which is polydopamine loaded with resiquimod (R848) carbon dots, along with PD-1 antibody therapy effectively inhibits liver and lung metastases in mice [69]. The tumour-targeting synergistic triple-combination treatment of chemotherapy, photoimmunotherapy, and fluorescence and photoacoustic dual-modality imaging was made possible by the development of a polypyrrole-loaded CPT-conjugated HA nanoparticle (P@CH). After further therapy with an anti-PD-L1 antibody, both the primary breast tumour and the lung metastasis were entirely eradicated in the mice [70]. Nanotechnology has the potential to advance therapeutic modalities, including photodynamic therapy and chemo-immunotherapy. Pheophorbide A, a PTX dimer prodrug (PXTK), and an anti-PDL1 peptide (dPPA) was administered using a hyaluronidase-responsive size-changing biomimetic nanoparticle (pPP-mCAuNCs@HA) coated in blood red cell membranes [71]. This combination approach creates many reactive oxygen species (ROS) after laser irradiation, which stimulates an anti-tumour immune response and increases the drug’s ability to reach the tumour’s deepest layers. Thus, the metastasis in the lung shrank to an undetectable level. Increasing the accumulation of Ce6 and chemotherapy has been proven to boost the anti-tumour immune response and prevent lung metastasis of breast cancer in animal models via shape-shifting NPs that simulate macrophages [72]. When biomimetic nano enzymes are used to target tumours, photodynamic immunotherapy and theranostics have a better chance of functioning synergistically. Manganese dioxide (MnO_2_) nanoparticles, for instance, have the potential to oxidise hydrogen peroxide (H_2_O_2_), leading to an increase in oxygen levels in hypoxic tumours and a decrease in metastasis [73]. To improve resistance to metastasis and the immune system, Liu et al. [74] created a kind of photodynamic therapy by combining an IDO inhibitor with the administration of a photosensitiser. Scientists came up with a redox-activated liposome nanovesicle approach based on porphyrin to slow tumour growth in mice, boost survival rates, and create strong anti-tumour metastasis therapy for breast cancer. There are both innate and adaptive immune systems. The monocytes, macrophages, dendritic cells, and natural killer cells that make up the innate arm of the immune system are the first line of defence against antigens. The cells of the innate immune system are responsible for identifying foreign cells and presenting antigenic cells to the cells of the adaptive immune system. Microorganisms, injured cells, and altered cells (such as tumour cells) may all be recognised by the receptors on the surface of innate immune system cells [75]. However, the T cells and B cells of the adaptive arm generate immunological memory, which lasts for much longer. The adaptive immune system relies on T and B lymphocytes, which, upon recognising an antigenic structure, multiply and then destroy it by triggering a variety of processes. Tissues and organs, such as the thymus, bone marrow, and spleen, as well as cytokines, antibodies, plasma, and adhesion molecules, make up the immune system.

Cancer can inhibit the immune system, allowing it to develop and spread without being detected. Therefore, the goal of cancer immunotherapy is to stimulate the immune system so that it attacks and destroys cancer cells [76]. Active or passive activation of the patient’s immune system towards the recognition and destruction of damaged or abnormal cells is the goal of immunochemotherapy. By controlling the tumour microenvironment and decreasing the amount of tumour neoantigen, cancer immunotherapy greatly aids in avoiding recurrence. Immune surveillance of cancer refers to the body’s attempt to identify cancer cells and early tumours. It takes the cooperation of numerous immune system cells for the body to recognise and eliminate damaged or cancerous cells. Tumour-associated antigens (TAAs) are the catalyst for this multi-stage process. Antigen-presenting cells (APCs) such as dendritic cells (DC), macrophages, and B lymphocytes deliver TAAs, which are discharged from dead tumour cells and occupy a large portion of the tumour surface, to T cells. Using Toll-like receptors (TLRs) and nucleotide-binding oligomerisation domain (NOD)-like receptors, DCs of the innate system identify pathogens, injured cells, and altered cells such as cancer cells. Tumour cells, like other immune cells such as T cells and MDSCs, may express TLRs. Both pathogen-associated molecular patterns and damage-associated molecular patterns are detectable by TLR-4 in a damaged cell. TLR-9, which is expressed in DCs and NK cells, is another factor that contributes to this field. TLR-9-based monotherapy and combo therapies are now being tested in clinical studies [77]. DCs modify their surface with antigens and present them to the T cell receptors via the class I and II major histocompatibility complex (MHC) in the lymph nodes, thereby presenting and activating the adaptive immune system cells. Class I MHC molecules may stimulate the production of cytotoxic T cells (Tc), which can eliminate tumour cells and release a greater quantity of tumour-associated antigens (TAAs) in the process. T helper (Th) CD4+ T cells may also recognise epitopes presented by the MHC molecule’s other part: the class II MHC molecule. The T cells that have recognised the tumour-specific epitopes and been activated and proliferated are known as effector T cells [78].

## 3. Nanotechnology-Assisted Targeting of Invasion/Intravasation

**Figure 3:**
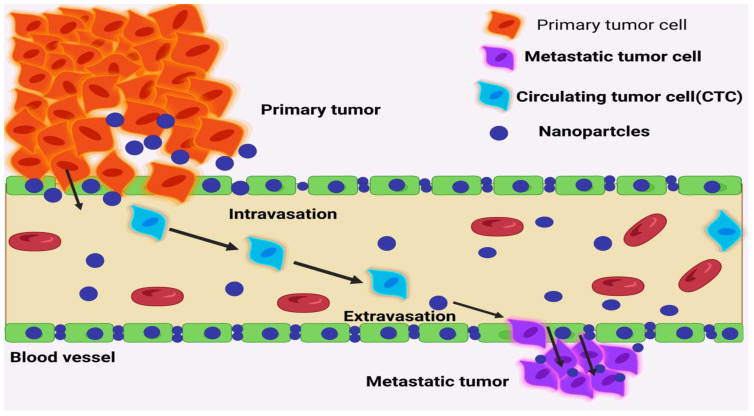
Diagrammatically representation of nanotechnology-assisted targeting of invasion/intravasation.

As a potential treatment for metastasis, scientists are examining strategies that aim to inhibit tumour cells’ aggressive invasion into nearby tissues and arteries. Combining Nanoquinacrine (NQC) with an inhibitor of activating adenosine monophosphatase (ADAM-17; GW280264) decreased the proliferation and invasion of cervical cancer stem cells [75]. Through this combined approach, the base excision repair mechanism was activated, which, in turn, reduced cancer metastasis. Figure 3 shows diagrammatically representation of nanotechnology-assisted targeting of inva-sion/intravasation. These findings suggest that Nectin-4, by sensitising cancer cells to the chemotherapeutic drug 5-FU, plays a crucial role in the resistance of metastatic cervical cancer cells to therapy. Using miR-125b-5p, a therapeutic and MRI-visible theranostic nanomedicine platform, has been found to be effective in targeting EMT and CSCs in hepatocellular carcinoma (HCC) [79]. A DOX-conjugated and nifuroxazide (NFX)-loaded micelle self-assembled with the aid of the B/pH dual-sensitive block copolymer developed by Luo et al.cathepsin., yielding a co-prodrug micelle (CLM). Testing CLM’s effects on mouse breast cancer cells in vitro revealed a decrease in cell viability and an interruption in the ability to metastasise and infiltrate. Treatment with the CLM molecule, given intravenously, improved anticancer and antimetastasis effects in a mouse model of breast cancer.

It takes a complicated series of actions for cells to undergo malignant transformation, whether due to genetic or epigenetic changes. Key characteristics of malignant cells are unchecked growth potentials and the capacity to infect neighbouring tissues and spread. Although the degree and timing of invasion and metastasis may vary according to the genetic and epigenetic heterogeneity within the tumour and additional signals from external variables, such as those within that specific microenvironment, cancer cells probably have some of these intrinsic capacities [80]. The majority of patients are expected to have micro- or macro-metastases by the time they seek medical treatment, despite much effort being put into early identification and prevention of cancer. Depending on their life expectancy, cancer patients in both the early and late stages are prone to metastasis. Over 90% of patient deaths related to solid tumours are caused by the metastatic spread of the underlying tumour. Despite this, research on metastasis lags behind other important activities such as proliferation and other processes.

This is caused by a combination of factors, including insufficient financing and research effort in this field as well as the intricacy of the metastatic process [81]. However, over the past ten years, there has been a significant advancement in this crucial area of cancer research. Nevertheless, there is still much that needs to be clarified before we can fully comprehend this nefarious condition, as well as several important gaps that must be filled. Even though there are few effective therapies available and significant morbidity and mortality are still linked to metastatic illness, the diagnosis and treatment of metastatic disease are crucial aspects of the ongoing fight against cancer that many patients must endure [82]. This supports and justifies more studies aimed at finding a less hazardous way to treat this illness, as do the complexity surrounding the metastatic process and the complex structure and heterogeneity of metastatic tumours. This is the main goal of understanding cancer. To develop novel approaches for treating metastasis, this study intends to examine significant information gaps and investigate prospective targets in combating metastasis and potential treatments, including phytochemicals, small molecule inhibitors, and natural substances [83].

## 4. Nanotechnology-Aided Targeting of Dissemination/Mobility and Migration

It is possible to halt the progression of metastasis by interfering with tumour cell migration and the binding of hetero aggregates to the endothelium. To treat tumours and prevent metastasis, Ding et al. [84] developed a nanoplatform that combines the iron chelator Di-2-pyridylketone-4,4-dimethyl-3-thiosemicarbazone (Dp44mT) with cisplatin in intracellular drug-accumulating as-NPs. The thiosemicarbazone chelator Dp44mT may be able to impede the movement and proliferation of tumour cells due to its high Fe-binding affinity and membrane permeability. Drug accumulation was improved in the NPs containing Dp44mT and cisplatin, while adverse effects were minimised and expression of HIF-1 and vascular endothelial growth factor was dampened. Therefore, the nanoplatform combination inhibited the development of orthotropic mammary tumours and decreased lung metastasis in mice with breast tumours. In this research, nanocarriers were used in conjunction with a variety of drugs, including chemo and iron chelators, to effectively stop the spread of tumours. Furthermore, it was discovered by Liu et al. [85] that the 100 nm iCluster platform could be diffused in breast tumours, shrinking from 50 to 5 nm in response to the acidity of the tumour. These nanoparticles have the potential to increase NP perfusion and transport from tumours to lymph nodes (LNs), thereby limiting lymph and lung metastases, all while delivering chemotherapeutic drugs to the circulatory system and circulating tumour cells (CTCs).

Cells that may migrate via blood vessels or lymphatic vessels are called CTCs. Cancer stem cells (CTCs) might be the focus of early cancer detection efforts, leading to real-time prognosis tracking and the creation of preventative measures against metastasis. For a comprehensive overview of CTC research using nanotechnology-enabled microfluidic devices, see Cheng et al. [86]. Nanoparticles with the right composition and size have the potential to slow the progression of metastatic disease (NPs). Carboxylated graphene oxide (CGO) was employed by Izadi et al. [87] in conjunction with trimethyl chitosan (TMC) and HA NPs containing HIF-1-siRNA and the CDK inhibitor Dinaciclib to downregulate HIF-1 and block CDKs in CD44-expressing cancer cells. Evidence shows that these nanoparticles have promising physicochemical features, including high cellular absorption, low toxicity, and low cytotoxicity (NPs). This combination treatment for cancer cells reduced tumour cell angiogenesis, proliferation, and colony formation by inhibiting CDKs/HIF-1 using this formulation. To back up these results, in vivo testing is required [88]. Improved lymphatic adherence of CTCs and reduced lung metastasis of breast cancer were seen in mice treated with biomimetic platelet membranes (PMNPs) or nanoplatelets coated with DOX and the FDA-approved photothermal agent ICG. Metastatic melanoma, breast, and pancreatic cancer cells were reduced in their ability to migrate and extravasate when exposed to C6 ceramide nanoliposomes [89]. A reorganization of the cytoskeleton, the dissolution of focal adhesions, and a change in integrin v3 affinity were the underlying processes. Zhu et al. [90] found that the anti-migratory and anti-invasive effects of the chemotherapeutic medication Etoposide given with LDH NPs (VP16-LDH) were greater than those of free VP16 in lung cancer cells in vitro. LyP-1 coating on these nanoparticles has been shown to specifically destroy the tumour and lymphatic endothelial cells. Lymph node absorption was enhanced via conjugation, which might be used to prevent cancer from spreading via the lymph system [91]. Preventing metastasis from happening in the first place might open new treatment avenues in the battle against cancer. EMT, microthrombi formation, and the construction of a pre-metastatic niche in mice with breast tumours were all inhibited by piceatannol and heparin as nontherapeutic H@CaPP [92]. Human translation studies assessing anticoagulants for targeted metastasis prevention should continue with caution due to the risk of bleeding complications. Gd-containing metallic fullerene nanoparticles reduce breast cancer stem cell (CSC) counts and HIF-1 expression in mice [93]. Using S100A4 siRNA conjugated to CBSA and coated with an exosome membrane, Zhao et al. [94] modified lung pre-metastasis niches in patients with breast cancer who had surgery. Strong gene silencing effects and a high affinity for pre-metastasis niche regions were shown by CBSA/siS100A4@Exosome, successfully restraining the formation of metastatic nodules. Peiris et al. [95] used a vascular targeting NP platform in conjunction with a theranostic strategy. In a mouse model of breast cancer metastasis, the feasibility of using GNPs to target micrometastases in artery beds was explored.

## 5. Nanotechnology-Supported Targeting of the Pre-Metastatic Niche and Micrometastasis

Seeding of tumours in distant organs requires a hospitable microenvironment rich in nutrients, extracellular matrix, and immune cells. The capacity of cancer cells to survive and spread in a given environment is connected to the nutrients present in that environment via the metabolic network. Premetastatic niche regulation may thus depend on limiting the availability of nutrients that facilitate metastatic seeding. Cancer cells are able to bind to the extracellular matrix, which then reactivates their survival signalling. In addition, fibronectin and hyaluronan, two components of the extracellular matrix, enable directed migration and boost metabolic activity. The intrinsic extracellular matrix of an organ is not always optimal for the attachment, metabolism, and migration of cancer cells that have been attracted to the site. As a result, extracellular matrix remodelling is a crucial step in the establishment of the premetastatic niche. Finally, the premetastatic niche is abundant in pro-tumour immune cells, which provide paracrine signalling support and anti-tumour immune cell suppression to facilitate cancer cell seeding. There seems to be a pre-existing environmental makeup in certain organs, such as the lungs, that is more conducive to the germination of cancer cells. Further, primary tumours actively shape the nutritional, extracellular matrix, and immune cell milieu of a distant organ before the entry of tumour cells, therefore generating a permissive and supportive premetastatic niche. An important part of the premetastatic niche environment includes immune cells that are pro-tumour. Thus, it has been shown that substances released from the original tumour attract neutrophils to the lung premetastatic niche [96].

**Figure 4:**
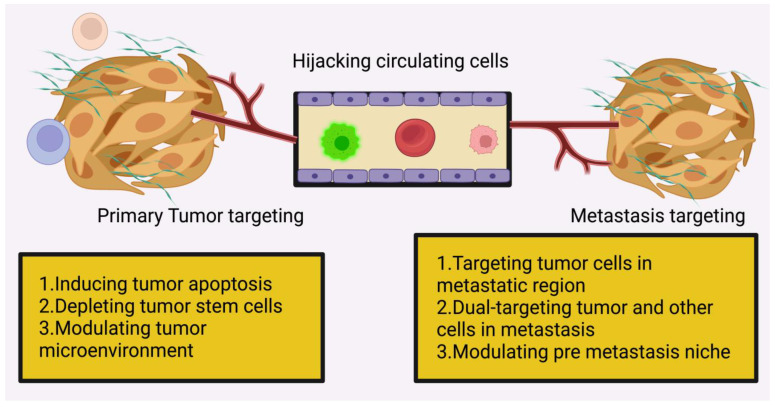
Schematic representation of perspective of nanoparticle-enabled tumour metastasis treatment.

In particular, it has been demonstrated that neutrophils establish an immune suppressive milieu by suppressing anti-tumour CD8+ T lymphocytes and that they boost the tumour-starting potential of cancer cells that arrive in the premetastatic niche via leukotriene signalling. Pro-tumour premetastatic niches often include macrophages, monocytes, and bone-marrow-derived cells despite the fact that patrolling monocytes may inhibit the effective seeding of cancer cells in the premetastatic niche. Recent research has shown that hepatic stellate cells (HSCs) are stimulated to produce fibronectin in response to pancreatic cancer exosomes, which are taken up by liver resident Kupffer cells. Macrophages produced from bone marrow are more easily recruited into this fibronectin-rich milieu. Figure 4 shows representation of perspective of nanoparticle-enabled tumour metastasis treatment. In addition, new evidence suggests that substances produced by tumours alter perivascular cells to create a pro-metastatic fibronectin-rich milieu. Liver premetastatic niches high in fibronectin, in turn, attract tumour-supportive macrophages from bone marrow.

In the premetastatic niche, fibronectin is not the only extracellular matrix component that is changed to attract pro-tumour stromal and immune cells. Similarly, the migration of myeloid cells to the lung premetastatic niche has been related to the activity of the enzyme lysyl oxidase (LOX), which crosslinks collagen of the extracellular matrix. Accordingly, LOX (lipooxygenase) may be secreted by primary breast tumours. Osteoclast-driven premetastatic lesion development in the bone may be mediated by extracellular matrix crosslinked by LOX activity [97], which is critical for the recruitment of pro-tumour immunological and stromal cells. As a result, there is a greater opportunity for tumour cells in circulation to seed and colonise into bone metastases. HIF1 stabilisation in osteoblast-lineage cells has been shown to affect the bone extracellular matrix, which, in turn, facilitates the metastasis of cancer cells to the skeleton. Cancer cells need certain nutrients in order to germinate in the premetastatic niche. For example, it has been established that breast cancer cells that have colonised the lung environment depend on pyruvate for energy, and they catabolise proline for their energy demands. Initiating metastases in oral cancer also requires the fatty acid receptor CD36. As a result, it is tempting to think that substances released by primary tumours also foster a nutrient-rich environment favourable to metastasis. Researchers have shown that miRNA122, which is released by tumours, affects the metabolism of local cells in the lung and brain, making more glucose available in the premetastatic niche to fuel the growth of newly arrived breast cancer cells. Together, released molecules from the main tumour set in motion establish the premetastatic niche, which increases the likelihood that newly arrived cancer cells will successfully seed the metastatic site.

## 6. Nanoparticle-Enabled (Macro) Metastasis Site Targeting Drug Delivery

**Figure 5:**
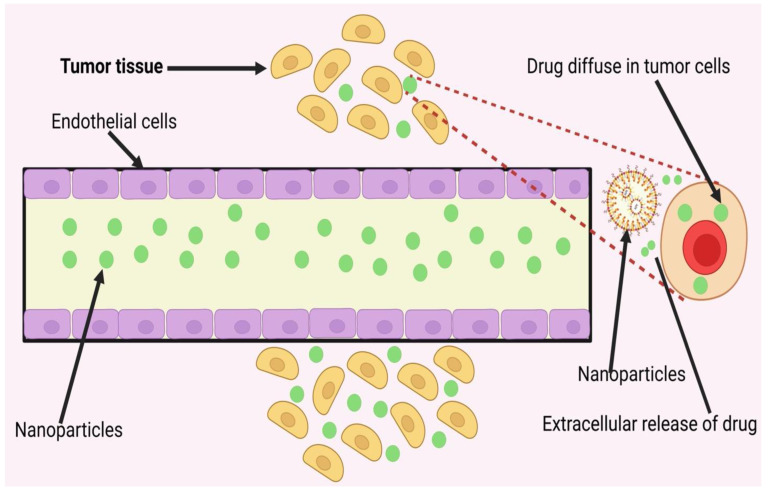
Schematic representation of nanoparticle-enabled (macro) metastasis site targeting drug delivery.

Several studies have shown that nanotechnology has the potential to be employed to successfully limit tumour metastasis by targeting tumour cells or TME at the metastatic site. In order to synthesise zoledronic acid, a third-generation nitrogen-containing bisphosphonate, Sun et al. used mesoporous silica NPs coated with gold nanorods (Au@MSNs-ZOL) (ZOL). Additionally, ZOL may cause osteoclast loss because it kills tumour cells, lowers VEGF levels, and blocks farnesyl pyrophosphate synthase. Injecting Au@MSNs-ZOL for photothermal therapy of bone metastases was more successful than using unmodified NPs (Au@MSNs) alone (achieved with laser light aid). Even in a rat model of intraosseous MDA-MB231 breast cancer cell injection, (Au@MSNs-ZOL + laser) was able to reduce bone metastases and pain [98]. The regularly used bisphosphonate alendronate (ALN-PAMAM) was used to adorn a dendrimer carrying the medication docetaxel (DTX@ALN-PAMAM). Both in vitro and in vivo experiments in mice found that this combination significantly decreased the size of bone metastases and the animals’ sensitivity to pain. The tibial bone lesion area was reduced, and bone volume fraction was increased in mice treated with alendronate-modified nanoparticles containing the Gli2 inhibitor alendronate. These effects were shown after the mice were given a small-molecule transcription factor loading (NPs). Figure 5. shows representation of nanoparticle-enabled (macro) metastasis site targeting drug delivery. 

**Figure 6:**
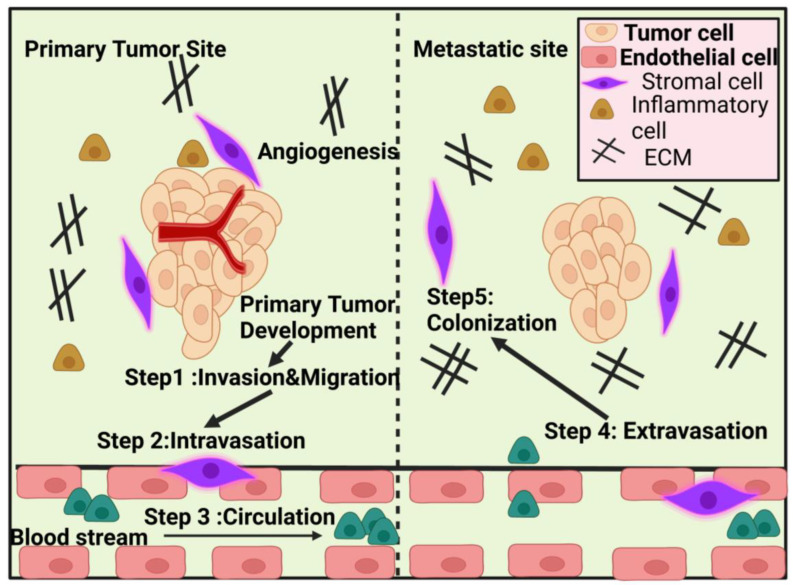
Schematic representation of primary tumour site and metastatic site in targeting metastases.

This enhanced medication’s reduced bone binding was a welcome side effect since it shortened the time it took for blood to circulate, a process known as nonphagocytic phagocytosis (NPHs). Considering that bisphosphonates only increased affinity with bone tissue and not tumour cells in bone metastasis, folic acid was conjugated to alendronate-modified PTX-loaded PLGA NPs to facilitate chemotherapy directed at both bone and tumour metastasis [99]. Folic-acid-treated nanoparticles enhanced the uptake of breast cancer cells in vitro. The reduced tumour growth rate and increased half-life of folic acid-modified NPs suggest that dual-targeting delivery may improve cancer cell absorption of PTX-loaded NPs for use in the treatment of bone metastases. In order to direct medicine to the site of metastasis, it may be possible to connect a homing protein such as LDH to it to make it multipurpose, as discussed above. The use of this method safeguards the DNA from the endonuclease and transports the DNA to the nucleus. The blood–brain barrier makes it challenging to provide medicines to the brain (BBB). Gene delivery NPs for targeted treatment of breast cancer brain metastasis were produced by Zhou et al. [100] using the modest pharmacologic antagonist of CXCR4 overexpressed in brain metastasis tumours, AMD3100. Promelitin, a cytolytic protein synthesised by these modified NPs, is released as melittin by tumour-expressed MMP-2, resulting in the death of cancer cells in mice. Promelittin-mediated gene therapy may be utilised to treat brain metastases of breast cancer, according to recent studies. When the primary tumour is sensitive to NP-drug conjugates (NDC), small-molecule drugs may be able to target brain metastases. Cornell prime dots (C’ dots) showed promise for delivering pharmaceuticals across the blood–brain barrier for the treatment of primary and metastatic central nervous system (CNS) illnesses, with high drug accumulation at the disease site and little off-target accumulation. Figure 6 shows representation of primary tumour site and metastatic site in targeting metastases.

Patients with primary gliomas or CNS metastases are now being studied in a Phase 1 imaging investigation looking at the expansion of ‘v integrin-binding cRGD-C’ dots. Biomimetic nanoparticles (NPs) may be an effective method for delivering medications that target metastasis because of their capacity to contact tumour metastatic cells selectively. For the treatment of breast cancer lymphatic metastasis, Ye et al. [101] reported the use of PLGA NPs coated with platelet membrane (PMNPs), also known as nanoplatelets, and loaded with DOX and ICG. Drug-loaded PMNPs reduced lung metastases when administered intravenously to mice, while platelet membrane coating boosted the lymphatic adhesion of CTCs. Reducing the unanticipated toxicity to normal cells in many metastatic nodules is possible and a much-needed strategy. Depleting metabolic energy was Huo et al.’s [86] strategy for eliminating liver cancer cells that had spread from other parts of the body. Nucleus-targeting W18O49 NPs (WONPs) were bound to a mitochondria-selective mesoporous silica NP (MSN)-bearing photosensitiser using a peptide that can be cleaved by the cathepsin B enzyme (Ce6). Malignant metastatic cells may be treated with photodynamic and photothermal therapy thanks to peptide linker decleavage, which spares countless healthy cells. Cancer-associated fibroblasts (CAFs) play a significant role in the TME and aid in the spread of tumours. There are many types of CAFs, such as primary tumour CAFs and metastatic fibroblasts, also known as “metastasis-associated fibroblasts” (MAFs). The metastatic tumour’s extracellular matrix (ECM) may be modified, immune cells in the tumour microenvironment (TME) may be modulated, angiogenesis may be induced, and malignant tumour features may be exacerbated by both drugs. The presence of MAFs may foster the formation of niches favourable to metastasis and the resistance of tumours to therapy. New blood vessel growth is promoted and resistance to anti-angiogenic therapy is increased when MAF activation raises tissue stiffness. Activated HSC/myofibroblasts, the liver cancer equivalent of CAF/MAF, might be targeted for drug delivery in the presence of NPs and nano ligands. Glucose-6-phosphate (M6P) or cyclic peptides that bind to the PDGFR receptor (PDGFR) are attached to the surface of nanocarriers. The distribution of medicine to activated HSC/CAF was aided by IGFRII. In vitro, siRNA was efficiently absorbed and delivered by activated HSC loaded with nano hydrogel particles and lipoplexes bearing the RNAi cargoSome kinds of hepatocellular carcinoma (HCC) and pancreatic cancer (PDAC) have been shown to respond to drugs that target stroma components such as circulating endothelial cells and hematopoietic stem cells (CAF/HSC). See Li et al. [88] for more details on the use of nanomedicine to halt the progression of stroma-rich pancreatic tumours. Yin et al. [102] built reduction-responsive polypeptide micelles from methoxy PEG block poly(S)-tart-Butadiene polymer copolymers as a means of regulating DOX dosing in osteosarcoma treatment. Micelle copolymers fared better than free DOX in terms of pharmacokinetics, tumour accumulation, and cardiac distribution. Micelles’ targeted tumour accumulation enhanced their anti-tumour and anti-metastasis properties while reducing their systemic toxicity. Although nanoparticles are often employed to deliver drugs that are specifically targeted for tumours, their limited penetration and retention sometimes result in unsatisfactory anticancer effects. We are all aware of the contradiction that although smaller sizes may penetrate deeply with better tumour retention, larger nanoparticles prefer to disperse around tumour blood vessels rather than enter the tumour parenchyma. Recently, a clever, size-tunable technique offered a solution to the nanoparticle size puzzle and showed promising application possibilities. In this study, we provide an overview of several stimuli-induced aggregation and shrinkage approaches for tumour-targeted drug delivery that may simultaneously boost retention and penetration of nanodrugs at tumour areas, hence enhancing therapeutic effectiveness. The morphology of the initial nanodrugs is altered by the introduction of internal (enzymes, pH, and redox) and exterior (light and temperature) stimuli via protonation, hydrophobisation, hydrogen bonding, stacking, and enzyme-resulted click reactions or dissociation, among other processes. Size-tunable techniques have several potential uses outside of oncotherapy, including real-time bioimaging and diagnostics, both of which are covered in this study. Finally, a full discussion of the prospects and probable application issues is provided, offering suggestions for further clinical change [103].

## 7. Limitations in the Current Metastasis Preclinical

Although some preclinical studies indicated that various medications had variable effects on main tumour vs. metastatic disease, the bulk of preclinical models to yet have focused on short-term decreases in primary tumour growth. In addition, we rely extensively on mouse models for preclinical data for metastatic investigations, even though their predictive ability is often decreased by severe limitations. Although some preclinical studies indicated that various medications had variable effects on main tumour vs. metastatic disease [104], the bulk of preclinical models to yet have focused on short-term decreases in primary tumour growth. In addition, we rely extensively on mouse models for preclinical data for metastatic investigations, even though their predictive ability is often decreased by severe limitations. Transgenic mice, in which individual genes are altered using recombinant DNA technology, may provide a more lifelike model of metastatic illness. In addition, each of the aforementioned model systems has a major drawback in that it often recapitulates just specific phases of the metastatic illness, making complete investigations difficult. Finally, most existing preclinical models do not investigate whether metastasis development and therapy could be affected by simulating the adjuvant scenario when the underlying tumour is not present [105].

### Limitations in the Current Clinical Setting

The length of this study and the number of participants needed make it challenging to organise clinical trials for medications aimed at metastasis prevention. In addition, standard research outcomes may not be achieved for drugs targeted for metastasis prevention since they are not necessarily meant to be cytotoxic or to successfully synergise with conventional chemotherapy regimens. Because of this, there has been a need for innovative trial designs. It has been suggested that primary prevention studies should focus on time to initial metastases or time to new metastases rather than tumour shrinkage in this metastatic scenario. Implementing further biopsies and cutting-edge imaging probes would also be necessary for these types of situations for proper disease progression monitoring [106].

## 8. Nanotechnology-Based Imaging of Metastasis, Sonodynamic Therapy, and Photoacoustic Imaging

Early diagnosis is the first step toward effective treatment of metastatic illness. This may be difficult to detect because of factors, such as its possible small size, lack of vascularity, and distance from the underlying tumour site. To enhance the visualisation of metastatic lesions throughout the body and the administration of therapeutic medications, NP-based imaging agents are utilised in combination with conventional imaging modalities. A further gain from nanotechnology is the development of nano theranostic techniques that make use of drugs for both diagnosis and therapy. Nanoparticles may be used to identify and localise metastatic cells, which are not easily visible using conventional imaging techniques. Magnetic resonance imaging (MRI), positron emission tomography (PET), and computed tomography (CT) are all examples of conventional imaging modalities that have made significant advances in cancer diagnosis. Nanoformulations have been developed for the detection of micro and macrometastases after breast cancer metastasis [107]. These nano-formulations, such as multifunctional superparamagnetic iron oxide NP (SPIO, SPION), combine Gd for MRI contrast and use antibodies or other specific ligands. It is possible to administer imaging and therapeutic substances that cannot cross the blood–brain barrier using surfactant coatings, such as the poly(methacrylic acid)-polysorbate 80-grafted-starch nano theranostic system. Brain metastases treatment using both prognostic functional imaging and differential MRI diagnosis. Prediction of melanoma brain metastasis may be improved using circulating tumour-infiltrating immune cells (SPIONs) that provide information on the tumour’s vasculature and heterogeneity. Iron oxide nanochain particles have been employed in fluorescence molecular tomography and multimodal imaging for detecting metastases of breast cancer in the lung, liver, and brain. A new generation of modified SPIO NPs has been developed due to its promise in theranostic applications. PET tracers were coupled to antibodies targeting metastasis-associated surface antigens including anti-carcinoembryonic antigen to detect and follow colorectal cancer metastases in the liver. Cornell prime dots (C’ dots) have previously been reported [108] as a dual-modality (PET-optical) strategy for administering native small molecule therapies to CNS metastases. Fluorescent and iodine-labelled C’ dots were used for multimodal imaging and monitoring of melanoma lymph node metastases. Orthotopic breast cancer models were used in a PET/CT investigation to evaluate the efficacy of [18F] tetrafluoroborate ([18F]BF4) in detecting metastases with excellent sensitivity and specificity. Due to its superiority as a reporter over the more traditional tracer [123I] iodide (sequential SPECT/CT), [18F]BF4 has been demonstrated to be more successful than [123I] iodide. This is because [18F]BF4 is rapidly absorbed by tumours and completely cleared from circulation. When real-time cell tracking in vivo is needed for preclinical research, this technique is recommended. The “polysome” form of these medications has also been developed for the treatment of bone metastases in animal models of prostate cancer [109]. Interventional radiologists used Gd-shell-coated Au nanorods loaded with theranostic NP (TNP) therapy for MR/X-ray contrast to treating colorectal cancer liver metastases. Metastasis heat damage was much amplified when TNPs were delivered to hepatocytes instead of the circulation [110]. Albumin-based theranostic nano-probe HSA-Gd-IR825 was paired with photothermal ablation of lymph node metastases after surgery to provide a molecularly targeted imaging strategy for malignant tumours. Combining W18O49 NPs with an anti-HER2 antibody might be useful for theranostic applications, including imaging lymphatic metastases from breast cancer and destroying them with photothermal therapy. In vivo, optical imaging requires brightly emitting, tissue-specific materials that can optically transmit through live tissue and be scanned with portable equipment that displays data in real-time. Noninvasive molecular imaging has the potential to improve our understanding of cancer development, macrometastasis, and tumour angiogenesis, as well as functional readouts of subcellular biological processes, including protein–protein interactions. Cancer dynamics in situ may be challenging to analyse owing to factors such as tissue absorption interference and auto-fluorescence. Nanocomposites of rare-earth NPs encased in human serum albumin (ReANC) have been used to monitor tumour growth and spread in melanoma animal models [111]. These very luminous Re nanoparticles have a substantially greater detection sensitivity than conventional short-wave infrared (SWIR) emitters, suggesting that they may improve the anatomical resolution of multispectral in vivo imaging. This enhances optical imaging in living organisms, which has applications in diagnostics and image-guided surgery. This study [112] shows that erbium-doped ReANCs, utilising a cocktail of niche-targeted probes with enhanced safety and clearance properties, can detect erbium ions and monitor multi-organ metastases in mice with basal human breast cancer. The lack of contrast between the sick lesion and the healthy tissue around it is one limitation of fluorophores. Re-albumin nanocomposites have been functionalised using methods such as using albumin nano shells to bind ligands selectively. Albumin nano shells improve contrast, cancer cytotoxicity, and collateral damage to healthy primary cells in a melanoma spheroid model [113]. A series of ICG-conjugated ultra-pH-sensitive polymeric micellar NPs was developed by Bennett et al. [114] to detect and surgically eradicate (micro)metastasis in LNs in an imaging-guided way in a rat breast cancer model. Zheng et al. [115] developed an iridium-based, hypoxia-activated, optical oxygen nano sensor with the intent of creating nanobots of the future. The micelle nano sensors demonstrated excellent biocompatibility and robust signals in metastatic tissue in cell and animal models. This hypoxia-activated nano sensor for cancer metastasis detection might be carried by the blood or lymph to the metastatic location. To create a biocompatible platform, the authors coated human haemoglobin with brilliant platinum nanoclusters (Pt NCs; Hb/Pt NCs). This setup has the potential to be utilised in the creation of a theranostic approach that zeroes in on CD44-overexpressing cancer cells and CSCs. Radiation-enhanced dual-ligand NP radionuclide imaging has also been utilised to identify metastatic breast cancer. Vascular targeting induced early-stage metastatic “hot spots” in the lungs of mice with breast cancer metastasis, as shown by gamma scintigraphy using Technetium-99 m (99mTc) as a radionuclide marker for NPs [116]. Using radionuclide imaging, scientists have shown that GNPs may be able to efficiently target micro metastasis in artery beds with a low dosage in a rat model of breast cancer metastasis. To do this, a v3-targeting ligand was attached to GNPs and labelled with 99mTc. One intriguing strategy for doing so is the use of radiation-sensitising nanoparticles (RNPs) for imaging and treatment on the same nano-object. Radio sensitisation of patients with melanoma, lung, colon, and breast cancer brain metastases before radiation was shown in a Phase 1 clinical study by Verry et al. [117]. Researchers showed that the advantages of using NPs in MRI were equivalent to those of using a therapeutic contrast agent that is already on the market. This theranostic NP medication has successfully progressed from preclinical to clinical application and is presently entering Phase 2 clinical trials.

Synthetic low molecular weight anilinoquinazolines include the first generation TKIs gefitinib (ZD1839; Iressa^®^, AstraZeneca, Cambridge, UK), erlotinib (Tarceva^®^, Genentech, San Francisco, CA, USA) and lapatinib (TYKERB^®^, GlaxoSmithKline, Brentford, UK). Anti-cancer efficacy against EGFR mutant tumours has been shown in large-scale clinical investigations in patients with NSCLC, which were inspired by positive findings from pre-clinical research [118].

Gefitinib was the first EGFR tyrosine kinase inhibitor to hit the market. Once only available in Japan, gefitinib has subsequently gained FDA approval as a frontline therapy for patients with EGFR mutations (exon 19 deletions or exon 21 L858R replacements) in metastatic NSCLC. According to results from the ‘IPASS’ clinical trials and the follow-up ‘IFUM’ studies, gefitinib significantly outperformed the gold standard treatment of carboplatin and paclitaxel in terms of median overall survival (18.6 vs. 17.3 months), median progression-free survival (24.9 vs. 6.7%; p. 0.001), and objective response rates (43.1 vs. 32.2%; p. 0.001) (Table 1). Almost half of patients saw their tumours shrink following therapy, with the effect lasting for around six months on average. Over 90 nations have now given gefitinib the green light. Although gefitinib’s anti-tumour effect has yet to be thoroughly characterised, it has been shown to decrease tyrosine kinase activity by competitively binding to the intracellular ATP-binding region of EGFR. In some patients with non-small cell lung cancer (NSCLC), gefitinib treatment has resulted in impressive and long-lasting responses, but in clinical studies of other cancers expressing high levels of EGFR, such as prostate, breast, head and neck, CRC, mesothelioma, brain, kidney, gastric, and ovarian cancers, the drug has shown very limited activity, if any. Interstitial lung disease, liver damage, gastrointestinal perforation, severe diarrhoea, and ocular abnormalities were identified as potential side effects of gefitinib in these clinical studies, in addition to the more prevalent adverse effects of diarrhoea and skin responses [119].

Ultrasound is a mechanical sound wave having a periodic vibration at a frequency greater than the 20-kilohertz threshold of human hearing. Sonic waves are produced by stimulating an ultrasonic transducer (often based on a piezoelectric component or an electromagnetic inductor) at the correct frequency, causing the transducer to translate the electrical signal into a mechanical displacement. The three main components of most ultrasound equipment are the generator, the compensating amplifier, and the transducer 

Ultrasonic waves are well known to have both thermal and non-thermal effects. When ultrasonic waves are absorbed by tissue, they compress and decompress the tissue mechanically, resulting in a temperature rise known as thermal effects. Part of this mechanical energy is wasted owing to friction effects, and it is transformed into heat. This results in alterations in membrane permeability and the fluidity of the phospholipid bilayer that makes up cell membranes in biological systems.

The non-thermal impact of ultrasound is a complex and varied collection of processes, encompassing steady and inertial cavitation, microstreaming and radiation forces. Microjets and microstreams are two types of these phenomena that may cause both thermal and mechanical stresses. In further depth, during non-inertial cavitation (also known as stable cavitation), the gas pockets present in the liquid fluctuate around an equilibrium radius and may survive numerous sonic compression and decompression cycles. Streaming fluid is produced by these oscillations, and the medium is mixed as a result of mechanical tensions. On the other hand, inertial cavitation occurs when ultrasound is used to cause gas bubbles trapped in a fluid to expand rapidly and then violently collapse. High pressures (more than 800 atm) and temperatures (greater than 5000 K) are generated during such a collapse, releasing a great deal of energy. The inertial cavitation is possible to cause water thermal dissociation and hence reactive oxygen species (ROS). In addition, a phenomenon known as sonoluminescence (SL) occurs when cavitation occurs [120].

Present-day medical practice makes extensive use of ultrasound for both diagnostic and therapeutic functions. The ultrasonic wave’s strength and frequency are both factors in producing the desired biological effects. It has cheap operating and instrumental expenses, is relatively safe for human health, and has a high tissue penetration potential. Sonography, which uses ultrasound, is a valuable imaging and diagnostic tool. For instance, the diagnostic focus of the ultrasonic waves is at a certain depth. The dispersed signal is recovered, enabling the image reconstruction of distinct tissues, due to their varying acoustic resistances. Microbubbles were created as contrast agents to increase the echogenicity and ultrasonic responsiveness of certain tissues. In a nutshell, they are a collection of gases that improve echogenicity, all of which are stabilised within a lipid or protein shell. This allows for the collection of 2D and 3D pictures of internal organs and tissues [121].

In addition, ultrasonography was employed to treat a wide variety of diseases, including soft tissue injuries, wound healing, edoema resolution, and scar tissue softening. In urology, lithotripsy treatments were used to remove stones, and therapeutic uses of low-intensity pulsed ultrasound were discovered for promoting bone formation. Standard procedures in cosmetic surgery for removing fat tissue include ultrasound-assisted lipolysis and liposuction [122]. However, they are not the focus of this study, and anyone interested may learn more by reading recent evaluations of gold.

This article will discuss the sonodynamic therapy of tumour cells or tissues using ultrasound in the presence of either soft or solid-state nanoparticles (NPs). (SDT). Very recently, a review of NPs and nanomaterials for SDT was published. Another recent study focuses more narrowly on the mechanics of SDT in experimental medicine and biology. In this work, we present an update to the state of the art in this area, with a particular emphasis on the processes behind the synergistic impact of NPs and acoustic fields towards the enhanced sonodynamic therapeutic result [123].

## 9. Translations of Nanomedicine in the Past and the Future

Within the past two decades, research into nanomedicine has skyrocketed. Findings from a PubMed search for “nanoparticle” show that the number of publications covering this area of study has gradually grown from its beginnings in the early 2000s. Others, for instance, have suggested that academic researchers do not have much relevance to the actual world. However, the FDA has frequently given the go-ahead to nanotechnology-based pharmaceuticals. Doxil (1995) and Abraxane have been superseded in cancer treatment by newer liposomal cytotoxic drug compositions [124] such as Marqibo, Onivyde, and Vyxeos (2005). Hensify (hafnium oxide nanoparticles for irradiating soft tissue sarcoma) and Nanotherm (particles for hyperthermia therapy of glioblastoma) have both been granted clinical use approval by the European Union [125]. Table 1 and the Supplementary Material of this publication, as well as de Lazaro and Mooney, Kemp and Kwon, and Ventola [126,127,128], provide a complete list of nanomedicines for cancer that have gained regulatory approval and are presently in clinical research. There was only little improvement in overall patient survival as a result of the use of these nanomedicines, which mitigated potentially fatal side effects of the therapy, because of the limitations of nanotechnology to improve survival or because of poor choice of disease targets, APIs, or translational methods. Since it was mistakenly believed that “one size fits all,” first efforts at translations concentrated on using “platform technologies” developed in engineering laboratories to treat a wide variety of cancers and other diseases. Most NP management systems did not have this feature. More and more designs focused on a certain therapeutic use raise doubts about it. In the early stages of developing new medical technology, risk aversion is a common concern [129]. To increase their chances of success, many of the many nanomedicines now in development depend on tried-and-true NPs (e.g., liposomes) and APIs (e.g., paclitaxel and doxorubicin). Since neither Nanotherm nor Hensify relies on NP’s supposed capacity to serve as a medication delivery vehicle, this is the first time such drugs have been made available to the public. New studies [130] suggest that nanomedicines with novel shapes that make use of the additional material features of NPs may be more effective. Comparable active pharmaceutical ingredients are employed, with the bulk of NPs used so far transporting small-molecule chemotherapeutic medicines. ONPATTROTM (patisiran, Alnylam Pharmaceuticals) was authorised in 2018 as an RNAi therapeutic agent for the treatment of polyneuropathy in amyloidosis, even though it is not a cancer therapy. It might expand the range of medicinal compounds that can be delivered by NPs. Possible ways to improve nanomedicines’ translational success and therapeutic use include the creation of companion diagnostics and the incorporation of imaging modalities into clinical trials. ‘Niche’ medical applications that can be handled by nanotechnology but do not currently have viable contemporary solutions should be identified, which will increase the success and utility of nanomedicines [131]. It seems that the original, probably too optimistic, aims for cancer nanotechnology translation have not been met. Nonetheless, they are evolving swiftly and are accountable for a great number of annual NDAs and approvals. Clinical research received 14% of all cancer nanotechnology funds during the last five years, according to an examination of the NIH grant database iSearch. When you search for “cancer” and “nanoparticle” on ClinicalTrials.gov, you will find 327 studies, 128 of which are presently recruiting participants (see Supplementary Material). Researchers have identified 70 Phase II trials and 14 Phase III trials if they omit Early Phase 1 and Phase I studies from the “already ongoing” search. This is not improbable if you are investigating a narrow field of study [132]. The investigators went over the authors’ introductory remarks to help frame the discussion. An examination of National Institutes of Health (NIH) cancer funding suggests it is difficult to find medicines that have shown success in clinical trials. It is not only nanotechnology but innovative medicines that are slow to make it into clinical practice. The mRNA-based Pfizer/BioNTech and Moderna COVID-19 vaccines administered through lipid nanoparticles have shown promising results [133]. Every year, almost 500 million doses of these two vaccines were administered in the United States alone. mRNA-based cancer therapies will become accessible shortly due to their efficacy as a delivery mechanism [134,135,136].

## 10. Future Perspective

Biomarkers for the metastatic potential that may direct medication development for chemoprevention are an exciting prospective future direction. Therapeutic efforts aiming to prevent metastatic spread in neoadjuvant as well as adjuvant settings will require the discovery of polymorphisms, genetic mutations (either germline or acquired), and plastic epigenetic alterations that determine susceptibility to metastasis (as opposed to metastasis-specific mediators). Understanding the biology of the complex interplay between tumours and their surrounding stroma, especially of interactions driven by plastic mechanisms, is necessary because these biomarkers may depend on mutational or gene expression profiles as well as microenvironmental factors within each malignancy. A growing amount of data suggests that microRNAs (miRNAs) play a crucial role in influencing malignant transformation and metastasis, in addition to epigenetic-driven plasticity. Most notably, members of the miRNA-200 family regulate EMT and have a role in inhibiting metastasis. While it is clear that the autocrine TGF-ß/ZEB/miR-200 regulatory axis regulates the plasticity of the transition between epithelial and mesenchymal states, it is also possible that other elements, such as the recently discovered competitive endogenous RNAs (ceRNAs), play a role in this process. In order to decipher the complexities of this interplay and direct the therapeutic use of these biological entities, it is expected that more sophisticated network-based techniques would be necessary. Despite the difficulties involved in getting miRNAs to where they need to be, two clinical studies are presently being conducted. Because of their distinct but pleiotropic impacts on a variety of characteristics associated with metastasis, miRNAs are a promising therapeutic target.

After metastatic dissemination has occurred, it is still possible to explore new treatment approaches. The best therapeutic options, either as single agents or in combination, before and after resection of the primary tumour are likely to be affected by an understanding of the impact that systemic modulation by primary tumours has on priming future metastatic niches and on influencing the growth of established metastases. As we have already shown, there are likely to be significant obstacles to keeping metastatic cells in a quiescent condition. Since quiescent cells are halted in their cell cycle progression, non-genetic factors are anticipated to play a key role in their revival. As a result, transforming metastatic illness into a chronic condition may be within reach if we can learn more about the molecular intricacies behind these pathways.

If we want to test effective and efficient true metastatic treatments, we need to make adjustments to the present clinical trial system. Evidence for effective antimetastatic medicines is mounting, suggesting a potential change in perspective. Reorganised clinical environments will benefit greatly from the incorporation of cutting-edge imaging technologies for tracking illness development and guiding treatment decisions. Recent developments in single-cell high-resolution fluorescence imaging, as well as in the recording of CT, MRI, and PET scans, are highly encouraging in this regard. These therapeutic efforts may be bolstered by further advancements in diagnostics. Particularly, the improvement of tests for detecting, collecting, and analysing circulating tumour cells (CTCs) should pave the way for highly dynamic, minimally invasive monitoring of single-cell genetic alterations. This approach has a wide range of potential therapeutic applications, including but not limited to the diagnosis of minimum residual illness, the monitoring of metastatic progression (using CTCs as biomarkers), and the discovery of novel targets in response to developed resistance.

Finally, it is not out of the question that new technology will alter how we approach stopping and treating metastasis. Recent advances in both preclinical and clinical contexts have been marked by the introduction of CRISPR/Cas9-based genome editing and immunotherapy methods. Genome-wide screening using the CRISPR/Cas9 technology in a mouse model of metastasis has uncovered loss-of-function mutations that contribute to tumour progression and metastasis. This potent genome editing technology has the potential to shed light on novel in vivo applications, such as the resensitisation of acquired resistance or the restoration of metastasis suppressor genes. Since immunotherapies, and, particularly, immune checkpoint therapy, have shown success in treating a wide variety of tumour types, it is possible that this method, alone or in combination, could be applied to the problem of eliminating metastatic cells that have entered immune-mediated dormancy.

## 11. Conclusions

Nanotechnology has unquestionably brought in a new era in the fight against and treatment of cancer. Many types of cancer have responded well to organic and inorganic NPs used in treatment. In addition to its benefits in pharmacokinetics, biocompatibility, tumour targeting, and stability, nanoparticle (NP)-based drug delivery systems have been found to minimise systemic toxicity and overcome drug resistance. As a result of these benefits, NP-based medications have been widely adopted for use in many areas of medicine, including chemotherapy, targeted treatment, radiation, hyperthermia, and gene therapy. Nanocarrier delivery methods may be useful in combating medication resistance caused by, among other things, the overexpression of efflux transporters, defects in the apoptotic pathway, and a hypoxic tumour microenvironment. NPs with a combination of cytotoxic and targeted molecules may be able to overcome drug resistance, albeit this will depend on the underlying mechanism of multidrug resistance. Studies show that hybrid nanoparticles have better delivery capacities; hence, they are becoming more popular. More in-depth studies on the biological features of different cancer kinds may enhance the direction of future pharmaceutical development efforts. The feasibility of employing hybrid NPs in cancer therapy, as well as the possibility of creating NPs that target cancer cells more selectively using targeting moieties, needs more study. Notable is the complicated relationship between NP and the immune system. Cancer drug resistance may arise from several different mechanisms, including drug inactivation, target shift, efflux, DNA damage repair, cell death inhibition, EMT, inherent cellular heterogeneity, epigenetic alterations, or a combination of these. As monotherapy increases the possibility of medication resistance, combination treatment is seen as the best therapeutic choice at the present time. Although Leslie Foulds first described the stages of metastasis more than 60 years ago, there are still few viable therapy choices for patients that aim to prevent or stop the metastatic process. Anti-metastatic medications are not yet part of the treatment arsenal against cancer, but the significant development witnessed in oncological research in recent decades gives hope for the future. Given the high levels of stress experienced by metastasising cells and the resistance of healthy tissues to the invasion of cancer cells, it is clear that the metastatic process is very inefficient. The goal of future metastasis research will be to identify the most targetable “Achilles heel(s)” in the cascade so that effective metastatic treatments may take advantage of them.

One especially exciting possibility is the identification of biomarkers for the metastatic potential that might direct medication development for chemoprevention. Therapeutic efforts aiming to prevent metastatic spread in neoadjuvant as well as adjuvant settings will require the discovery of polymorphisms, genetic mutations (either germline or acquired), and plastic epigenetic alterations that determine susceptibility to metastasis (as opposed to metastasis-specific mediators). Understanding the biology of the complex interplay between tumours and their surrounding stroma, especially of interactions driven by plastic mechanisms, is necessary because these biomarkers may depend on mutational or gene expression profiles as well as microenvironmental factors within each malignancy. MicroRNAs (miRNAs) are increasingly thought to have a crucial role in influencing malignant transformation and metastasis, in addition to epigenetic-driven plasticity. One of the most notable examples is how miRNAs from the miRNA-200 family regulate EMT and work to inhibit metastasis. It has been shown that the autocrine TGF-ß/ZEB/miR-200 regulatory network controls the plasticity of switching between epithelial and mesenchymal states, but it is clear that this axis represents only a small part of a much larger network that may include the activity of modulatory elements such as the recently discovered competitive endogenous RNAs (ceRNAs). In order to understand the intricate interplay between these biological entities and to exploit them therapeutically, more cutting-edge network-based techniques will likely be required in the future. Despite the difficulties involved in getting miRNAs to where they need to be, two clinical studies are presently being conducted. Because of their distinct but pleiotropic impacts on a variety of characteristics associated with metastasis, miRNAs are a promising therapeutic target. After metastatic dissemination has occurred, it is still possible to explore new treatment approaches. The best therapeutic options, either as single agents or in combination, prior to and after resection of the primary tumour are likely to be affected by an understanding of the impact that systemic modulation by primary tumours has on priming future metastatic niches and on influencing the growth of established metastases. As we have already shown, there are likely to be significant obstacles to keeping metastatic cells in a quiescent condition. Dormant cells, which are stalled in their cell cycle phase 5, are most likely to be reawakened by non-genetic means. As a result, transforming metastatic illness into a chronic condition may be within reach if we can learn more about the molecular intricacies behind these pathways.

If we want to test effective and efficient true metastatic treatments, we need to make adjustments to the present clinical trial system. A paradigm change may be required as evidence mounts in favour of effective antimetastatic medicines. Reorganised clinical environments will benefit greatly from the incorporation of cutting-edge imaging technologies for tracking illness development and guiding treatment decisions. Recent developments in computed tomography (CT), magnetic resonance imaging (MRI), and positron emission tomography (PET) imaging recording of single cells are highly encouraging in this regard. These therapeutic efforts may be bolstered by further advancements in diagnostics. Single-cell, minimally invasive monitoring of genomic alterations may be made possible by the improvement of tests for the detection, collection, and analysis of circulating tumour cells (CTCs). This method has potential therapeutic applications, including monitoring metastatic progression (using CTCs as biomarkers) and identifying novel targets in response to acquired resistance.

Finally, it is not out of the question that new technology will alter how we approach stopping and treating metastasis. Recent advances in both preclinical and clinical contexts have been marked by the introduction of CRISPR/Cas9-based genome editing and immunotherapy methods. Loss-of-function mutations that promote tumour development and metastasis were identified using a genome-wide screen using the CRISPR/Cas9 technology in a mouse model of metastasis. This strong genome-editing technology has the potential to be used in vivo to find novel ways to restore metastasis suppressor genes or resensitise against acquired resistance, among other uses. Immunotherapies, particularly immune checkpoint therapy, have shown success in treating a wide variety of tumour types, suggesting that this strategy, alone or in combination, could be used to eliminate metastatic cells that have entered immune-mediated dormancy.

## Figures and Tables

**Table 1:** Regulated nano-formulations of cancer medications.

Product Name	Composition	Indications	First Approval
SMANC	Polymer conjugate neocarzinostatin	Liver and renal cancer	Japan (1993)
Doxil/Caelyx	PEGylated liposomal doxorubicin	Myeloma, Kaposi’s sarcoma, and ovarian and metastatic breast cancer. Europe (1996)	US (1995)
DaunoXome	Liposomal daunorubicin	Kaposi’s sarcoma	US (1996)
DepoCyt	Liposomal cytarabine	Lymphoma and leukaemia	US (1999)
Myocet	Liposomal doxorubicin	Metastatic breast cancer	Europe/Canada
Abraxane	Albumin-bound paclitaxel	Non-small-cell lung and metastatic	US (2005)
Oncaspar	L-asparaginase conjugate	Breast and pancreatic cancer. Europe (2008)	US (2006)
Hensify (NBTXR3)	Hafnium oxide nanoparticle	Acute lymphoblastic leukaemia	EU (2019)
Vyxeos	Cytarabine/Daunorubicin	Locally advanced soft tissue	2017
Onivyde	Irinotecan	Sarcoma (STS)	2015
NanoTherm	Iron oxide nanoparticle	Acute myeloid leukaemia	EU (2010)
Mepact	Mifamurtide MTP-PE	Pancreatic cancer	EU (2009)

**Table 2:** Advantages and disadvantages of different nanostructures as carriers.

Drug Carrer	Advantage	Disadvantage
Metal Nanoparticles	Multifarious applications due to high surface area	Metal toxicity, stability, storage
Gold nanoparticles	Enhanced distinction, minimally invasive, no photobleaching	Toxicity, optical signal not strong, biocompatibility
Carbon- derived	High loading capacity, vast numbers of possibilities for surface modification	Few in vivo studies developed
Exosomes	Reduced immune response, protection of circulating genetic material, possibility of cell targeting	Limited transfection efficiency

## Data Availability

Not applicable.
