# Correction: Singh et al. Nanotechnology-Aided Advancement in Combating the Cancer Metastasis. *Pharmaceuticals* 2023, *16*, 899

**DOI:** 10.3390/ph17070954

**Published:** 2024-07-17

**Authors:** Arun Kumar Singh, Rishabha Malviya, Bhupendra Prajapati, Sudarshan Singh, Deepika Yadav, Arvind Kumar

**Affiliations:** 1 Department of Pharmacy, School of Medical and Allied Sciences, Galgotias University, Greater Noida 203201, India; arunthakur01996@gmail.com (A.K.S.); deepika.21smas2020010@galgotiasuniversity.edu.in (D.Y.); 2 Shree S. K. Patel College of Pharmaceutical Education and Research, Ganpat University, Kherva 384012, India; 3 Department of Pharmaceutical Sciences, Faculty of Pharmacy, Chiang Mai University, Chiang Mai 50200, Thailand; 4 Chandigarh Engineering College, Jhanjeri, Mohali 140307, India; arvindkumar.j1753@cgc.ac.in

## Addition of Reference

In the original publication [1], “Nanotechnology-Aided Advancement in Combating the Cancer Metastasis”, Reference [136] (see [2]) “Avula, L.R.; Grodzinski, P. Nanotechnology-aided advancement in the combating of cancer metastasis. *Cancer Metastasis Rev.* **2022**, *41*, 383–404. https://doi.org/10.1007/s10555-022-10025-7” was not cited. The citation has now been inserted into Section 9 “Every year, almost 500 million doses of these two vaccines were administered in the United States alone. mRNA-based cancer therapies will become accessible shortly due to their efficacy as a delivery mechanism [134–136]”. The authors state that the scientific conclusions are unaffected. This correction was approved by the Academic Editor. The original publication has also been updated.
